# Quantitative EEG may predict weaning failure in ventilated patients on the neurological intensive care unit

**DOI:** 10.1038/s41598-022-11196-7

**Published:** 2022-05-04

**Authors:** Tamara M. Welte, Maria Gabriel, Rüdiger Hopfengärtner, Stefan Rampp, Stephanie Gollwitzer, Johannes D. Lang, Jenny Stritzelberger, Caroline Reindl, Dominik Madžar, Maximilian I. Sprügel, Hagen B. Huttner, Joji B. Kuramatsu, Stefan Schwab, Hajo M. Hamer

**Affiliations:** 1 Department of Neurology, University Hospital Erlangen, Schwabachanlage 6, 91054 Erlangen, Germany; 2 Department of Neurology, University Hospital Giessen, Klinikstrasse 33, 35385 Gießen, Germany

**Keywords:** Neuroscience, Health care, Neurology

## Abstract

Neurocritical patients suffer from a substantial risk of extubation failure. The aim of this prospective study was to analyze if quantitative EEG (qEEG) monitoring is able to predict successful extubation in these patients. We analyzed EEG-monitoring for at least six hours before extubation in patients receiving mechanical ventilation (MV) on our neurological intensive care unit (NICU) between November 2017 and May 2019. Patients were divided in 2 groups: patients with successful extubation (SE) versus patients with complications after MV withdrawal (failed extubation; FE), including reintubation, need for non-invasive ventilation (NIV) or death. Bipolar six channel EEG was applied. Unselected raw EEG signal underwent automated artefact rejection and Short Time Fast Fourier Transformation. The following relative proportions of global EEG spectrum were analyzed: relative beta (RB), alpha (RA), theta (RT), delta (RD) as well as the alpha delta ratio (ADR). Coefficient of variation (CV) was calculated as a measure of fluctuations in the different power bands. Mann–Whitney U test and logistic regression were applied to analyze group differences. 52 patients were included (26 male, mean age 65 ± 17 years, diagnosis: 40% seizures/status epilepticus, 37% ischemia, 13% intracranial hemorrhage, 10% others). Successful extubation was possible in 40 patients (77%), reintubation was necessary in 6 patients (12%), 5 patients (10%) required NIV, one patient died. In contrast to FE patients, SE patients showed more stable EEG power values (lower CV) considering all EEG channels (RB: *p* < 0.0005; RA: *p* = 0.045; RT: *p* = 0.045) with RB as an independent predictor of weaning success in logistic regression (*p* = 0.004). The proportion of the EEG frequency bands (RB, RA RT, RD) of the entire EEG power spectrum was not significantly different between SE and FE patients. Higher fluctuations in qEEG frequency bands, reflecting greater fluctuation in alertness, during the hours before cessation of MV were associated with a higher rate of complications after extubation in this cohort. The stability of qEEG power values may represent a non-invasive, examiner-independent parameter to facilitate weaning assessment in neurocritical patients.

## Introduction

Weaning from mechanical ventilation (MV) usually follows standardized protocols on intensive care units (ICUs)^1,2^. Nevertheless, finding the optimal time point for extubation is crucial and often challenging^3^. Delayed extubation can cause pulmonary damage, carries a higher risk of ventilator-induced pneumonia and prolongs ICU stay^4–6^. Premature cessation of MV leads to aspiration, hypoxia and possibly a need for reintubation, which is associated with risk for complications^7^.

The establishment of standard criteria successfully facilitates weaning and extubation assessment. Typical criteria usually target cardiorespiratory function, e.g. by performance of spontaneous breathing trials (SBT) or investigation of the rapid shallow breathing index (RSBI)^8^. In addition, cooperativeness of the patient plays an important role, implying that the patient should be awake and follow commands^9^. Many studies revealed good prediction of successful extubation (SE) applying these criteria in the general critical care setting^10^. As a consequence, a low rate of extubation failure (EF) ranging between 5 and 10% is considered acceptable in ICU patients^11^.

Unfortunately, those widespread criteria cannot easily be extrapolated to neurocritical patients. EF rates in neurological intensive care unit (NICU) studies are reported to range up to 40%^12–14^. Moreover, NICU patients show longer duration of ventilation and have higher rates of ventilator-induced pneumonia and mortality compared to general ICU populations^15–17^.

Thus, applying standard assessment seems not as effective in neurological and neurosurgical patients^18,19^. An altered state of consciousness, brainstem lesions and functional neurological deficits may be the main reasons for this dilemma^14,17^. Patients suffering from brain pathologies usually are not ventilated because of a primary respiratory failure but due to impaired consciousness and respiratory drive^16^. Consequently, sufficient and stable vigilance and adequate awareness, usually required for extubation, are less frequent to observe and subject to fluctuations over time. In addition, typical neurological disorders such as aphasia and paralyses challenge sufficient evaluation of the patients who may not be able to follow commands or to move, which is often required to obtain the usual parameters of MV withdrawal^20^.

However, EEG which can record brain activity of large areas of the cortex is able to identify different levels of vigilance on the one hand and brain dysfunction on the other hand. Continuous EEG can easily monitor these activities over time^21^. Furthermore, quantitative EEG analysis (qEEG) represents a more objective and increasingly available tool for monitoring brain function on ICU and is already applied to several indications on NICUs including coma and subarachnoid hemorrhage^22^. Thus, EEG might also provide valuable additional information regarding the clinical question if a NICU patient has sufficient and stable vigilance and cerebral status to be a candidate for successful extubation.

Therefore, the aim of this observational prospective study was to identify qEEG parameters that are able to predict extubation success in neurocritical patients. We hypothesized that patients with and without successful extubation either show different composition of the qEEG power spectrum or varying stability in the qEEG frequency bands in the hours before extubation.

## Methods

### Patients and clinical management

All adults admitted to the neurological intensive care unit (NICU) at the University-Hospital Erlangen between November 2017 and May 2019 receiving mechanical ventilation were screened for prospective enrollment, regardless of the underlying pathology. For inclusion the following criteria had to be met: age ≥ 18 years, MV for at least 6 h, extubation in curative intention, availability and feasibility of EEG monitoring for at least 6 h within the last 12 h before extubation.

The clinical monitoring of the patients included systolic, diastolic, and mean arterial blood pressure, heart rate, temperature, oxygen saturation, and where necessary intracranial pressure (ICP). Neurological status was surveyed at least three times per day by NICU staff.

The weaning and extubation procedure followed a certified institutional standard operating procedure (SOP) according to the current international guidelines and was not influenced by study measures^8,23^. Required parameters for extubation were: stable ventilation in CPAP (continuous positive airway pressure) mode, ASB (assisted spontaneous breathing) support (Psupp) ≤ 12 mbar, and PEEP (positive end-expiratory pressure) = 5 mbar, successful SBTs (routinely performed three times per day with respect of cessation criteria). Required clinical parameters included: Sufficient and stable vigilance as judged by the NICU stuff in charge, sufficient cough/tracheal reflex.

Patients were divided in two groups: 1) patients with successful extubation (SE) without need for new ventilation support during the further hospital stay and 2) patients with complications after MV withdrawal (failed extubation, FE), including reintubation, need for non-invasive ventilation (NIV) or death after extubation. Decision on post extubation NIV was made individually and based on interdisciplinary consensus of NICU stuff.

When the EEG recording was performed because of clinical reasons such as status epilepticus or monitoring encephalopathy, the acquired EEG was also used for this study. In addition, the EEG monitoring was initiated for study reasons in the remainder of the patients fulfilling the inclusion criteria. The study was performed in accordance with relevant guidelines and regulations and was approved by the local ethics committee of the Friedrich-Alexander University Erlangen-Nürnberg. Written informed consent was provided by legal representatives in all patients receiving additional EEG monitoring for study reasons.

### EEG monitoring

In this study, we analyzed EEG in a simplified bipolar montage, which is safe and easy to apply as shown by several previous studies and reveals meaningful results in encephalopathic patients^24,25^. If patients received the EEG monitoring solely because of study reasons, we only applied this reduced electrode set, including ten standard scalp electrodes fixed with collodion according to the International 10/20 System leading to following channels: F4–C4, T4–P4, P4–O2; F3–C3, T3–P3, P3–O1^24,25^. In these cases, the continuous simplified EEG monitoring was started as soon as weaning from MV was planned. These recordings were not interpreted by the treating physicians. If patients were monitored for clinical reasons with the full set of electrodes according to the International 10/20 System, we offline reformatted the EEG to obtain the reduced montage to receive homogeneous EEG data in all patients.

EEG data were digitized at a sampling rate of 250 Hz with a high-pass filter of 0.05 Hz and a low-pass filter of 100 Hz (Carefusion EEG-system; Natus Mediacal Inc).

### Quantitative EEG analysis

Quantitative EEG analysis was performed for the last 6 h of EEG within 12 h before extubation. The unselected, continuous raw EEG signal underwent automated artefact rejection, which was evaluated and already used in previous studies^24,26,27^. Following artefact rejection, EEG power spectral analysis was performed, which consisted of a Fast Fourier transformation of 2-s epochs of EEG.

In the context of our study, relative EEG power values seemed more suitable than absolute values, because absolute power values vary considerably among individuals and make interindividual comparisons difficult. Moreover, relative power values are commonly used in qEEG studies e.g. for evaluation of delirium patterns^28,29^.

We determined the 1-min power values of the relative beta proportion of the global spectrum (RB, 12.5–22 Hz), relative alpha (RA, 8–12 Hz), relative theta (RT, 4–7.5 Hz), and relative delta (RD, 0.5–3.5 Hz). In addition, the alpha delta ratio (ADR) was calculated. Comparison of SE patients to FE patients was performed, focusing on the composition of the EEG power spectrum as well as on its stability over time. Therefore, we calculated the mean and standard deviation (SD) of all 1-min power values of the whole 6 h of EEG recording. To compare the extent of occurring fluctuations in the different power bands among patients, we normalized EEG power by dividing every single 1-min power value by the mean and accordingly determined the coefficient of variation (CV = ratio of the standard deviation to the mean), representing a relative measure of EEG power variability^30^.

The EEG parameters were analyzed for global power values over all channels and in different brain regions separately (fronto-central (F4–C4, F3–C3), temporal (T4–P4, T3–P3) and parieto-occipital (P4–O2, P3–O1)).

### Statistics

We performed Mann–Whitney-U tests to analyze differences in proportion of power values and the extent of power value fluctuations (using CV) between SE patients and FE patients. Due to multiple testings we corrected levels of significance applying the Benjamini–Hochberg method (False Discovery Rate)^31^. A corrected p value of ≤ 0.05 was considered statistically significant. Additionally, we applied multivariate logistic regression to identify independent factors for weaning success or failure. We also performed receiver-operator-characteristic (ROC) statistics and calculated the Youden index to identify the optimal threshold of occurring power fluctuations for the differentiation between successful extubation and extubation failure.

### Ethics approval and consent to participate

The study was approved by the local ethics committee of the Friedrich-Alexander University Erlangen Nürnberg. Written informed consent was provided by legal representatives in all patients receiving additional EEG monitoring for study reasons.

## Results

52 patients were included (26 (50%) male, mean age 65 ± 17 years, diagnoses: 40% seizures/status epilepticus, 37% ischemia, 13% intracranial hemorrhage, 10% others, Table 1). Median duration of mechanical ventilation was 132 h (range 10–400 h). All patients performed successful SBTs and fulfilled clinical criteria before MV withdrawal. Extubation was successful in 40 patients (77%). Among the remaining 12 FE patients (23%), reintubation was necessary in 6 patients (12%). Five patients (10%) required NIV. One patient died after ‘curative’ extubation because reintubation became necessary but was not aimed for according to the patient´s and relatives` will. The mean delay of re-initiation of ventilation support was 28.7 ± 28.1 h.

**Table 1:** Patients´ characteristics.

	SE patients (N = 40)	FE patients (N = 12)	*p* values
Age, mean (SD)	66 (15)	63 (19)	*p* = 0.713
Female, N (%)	20 (50)	6 (50)	*p* > 0.999
Diagnosis, N (%)			*p* = 0.542
Seizures	16 (40)	5 (42)	
Ischemia	16 (40)	3 (25)	
Intracranial hemorrhage	4 (10)	3 (25)	
Others	4 (10)	1 (8)	
Days of EEG monitoring (median, range)	4.5 (1–19)	6.5 (2–12)	*p* = 0.078
Hours of MV (median, range)	90 (10–400)	285 (50–340)	**p = 0.007**
Respiratory parameters			
PaO2 before extubation (mmHg, mean, SD)	94 (19)	104 (32)	*p* = 0.399
PaO2/FiO2 ratio (mean, SD)	319 (87)	349 (149)	*p* = 0.624
ASB Support (mbar, mean, SD)	3.3 (2.4)	2.6 (2.0)	*p* = 0.452
RSBI before extubation (mean, SD)	34 (13)	37 (16)	*p* = 0.768
Circulatory parameters			
Heart rate (mean, SD)	81 (15)	81 (20)	*p* = 0.881
Hemoglobin level (mean, SD)	11.0 (1.8)	9.9 (1.6)	*p* = 0.352
Clinical parameters			
RASS score (median, range)	− 1 (− 3–2)	− 0.5 (− 2–0)	*p* = 0.928
Focal neurological sings, N (%)	17 (42.5)	5 (41.7)	*p* > 0.999

ASB = assisted spontaneous breathing, FE = Failed extubation, FiO2 = fraction of inspired oxygen, MV = mechanical ventilation, mbar = millibar, mmHg = Millimeter of Mercury, N = number, PaO2 = arterial partial pressure of oxygen, RASS = Richmond Agitation Sedation Scale, RSBI = Rapid shallow breathing index, SD = standard deviation, SE = Successful extubation.

Significant values are in [bold].

In 11/12 patients (91.7%) fluctuations in vigilance were documented as a complication contributing to extubation failure. In one of these patients, fluctuating vigilance could be attributed to recurrence of seizures accompanied by aspiration and pneumonia leading to re-intubation. In another two of these patients, additional dysphagia and suspected consecutive aspiration were documented early after extubation. In one patient, an isolated dysphagia and insufficient oxygenation were documented as main reasons for reintubation, which had to be re-initiated after 6 h. Main reason for post-extubation NIV was insufficient oxygenation.

SE and FE patients did not differ significantly in age or gender. Diagnoses showed a similar distribution in both groups (*p* > 0.05). In addition, MV and cardiopulmonary parameters, as well as clinical evaluation of sedation and neurological function at the time of extubation did not differ in both groups (Table 1). Yet, in univariate testing, the median duration of mechanical ventilation was shorter in SE patients (90 h, range 10–400) in comparison to FE patients (285 h, range 50–340, p = 0.007).

EEG monitoring was maintained for a mean duration of 5 days (range 1–19 days, Table 1). Administration of sedative agents (e.g. midazolam, propofol) during EEG monitoring before extubation was similarly observed in both groups (SE: 19/40 patients (48%), FE: 8/12 patients (67%), *p* = 0.33).

The comparison of the proportion of EEG frequency bands of the global power spectrum did not show any significant differences between SE and FE patients for relative beta (RB, 12.5–22 Hz), relative alpha (RA, 8–12 Hz), relative theta (RT, 4–7.5 Hz) or relative delta (RD, 0.5–3.5 Hz) (*p* > 0.05). Neither was the ADR significantly different. This was true considering all channels or different brain regions separately.

**Figure 1:**
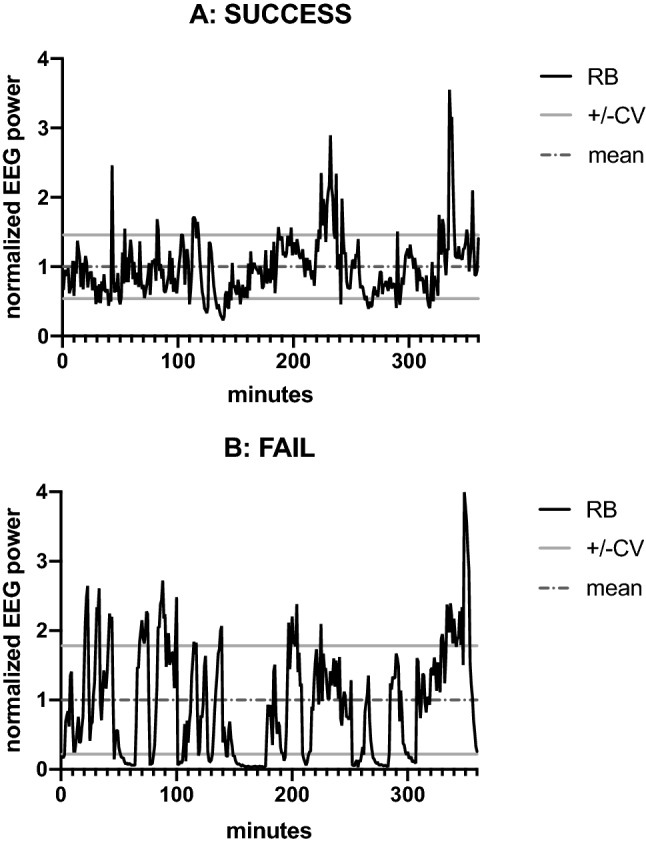
Examples of normalized relative beta EEG power values over time in two patients before extubation. (**A**) Patient 52, diagnosed with status epilepticus, successful extubation after 107 h of MV, CV = 0.46. (**B**) Patient 48, diagnosed with intracerebral hemorrhage in the left hemisphere, extubation after 156 h of MV, afterwards need for NIV and after 2 days need for reintubation because of insufficient vigilance and hypoxemia, CV = 0.78. CV = coefficient of variation, EEG = electroencephalogram, MV = mechanical ventilation, NIV = non-invasive ventilation, RB = relative beta.

However, the stability of the EEG power before extubation revealed significant differences between SE and FE patients. Successfully extubated patients showed more stable EEG power values reflected by lower CV measures than patients with complications after discontinuation of MV (Fig. 1). This was seen in the beta (*p* < 0.0005), alpha (*p* = 0.045) and theta frequency band (*p* = 0.045, Table 2). This effect could be observed especially in frontocentral EEG channels (Table 2).

**Table 2:** Median coefficients of variation (CV) of EEG power frequency bands in patients with successful extubation (SE) versus extubation failure (FE).

Frequency bands	CV in SE patients (N = 40)	CV in FE patients (N = 12)	Corr *p* value
Over all channels Relative beta Relative alpha Relative theta Relative delta Alpha delta ratio	61 (55–70) 39 (37–46) 31 (29–38) 8 (8–15) 48 (47–57)	92 (80–110) 48 (43–61) 41 (34–44) 11 (8–15) 62 (52–79)	** < 0.0005** **0.045** **0.045** 0.405 0.06
Fronto-central channels Relative beta Relative alpha Relative theta Relative delta Alpha delta ratio	72 (67–86) 45 (41–51) 35 (32–40) 10 (10–16) 57 (53–65)	97 (85–114) 56 (49–65) 50 (41–55) 13 (10–19) 75 (620–81)	**0.008** **0.035** **0.008** 0.307 0.051
Temporal channels Relative beta Relative alpha Relative theta Relative delta Alpha delta ratio	67 (64–83) 43 (42–52) 33 (33–43) 11 (10–16) 55 (54–65)	102 (84–123) 55 (46–66) 43 (38–48) 14 (10–17) 70 (55–87)	**0.015** 0.133 0.133 0.459 0.204
Parieto-occipital channels Relative beta Relative alpha Relative theta Relative delta Alpha delta ratio	56 (57–75) 47 (44–53) 38 (35–47) 8 (8–11) 55 (54–69)	74 (61–95) 64 (45–70) 39 (36–50) 8 (6–13) 73 (53–91)	0.363 0.363 0.363 0.919 0.363

Corr. *p* value = corrected *p* value by Benjamini&Hochberg (False Discovery Rate), CV = coefficient of variation, FE = Failed extubation, SE = Successful extubation; univariate testing using Mann–Whitney U test, all values in %, numbers in brackets indicate 95% confidence interval.

Significant values are in [bold].

**Figure 2:**
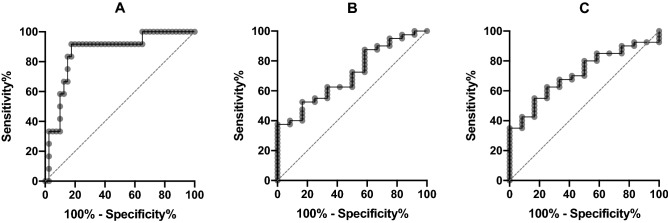
ROC curves of differences in EEG power value fluctuations over all channels in SE and FE patients. (**A**) relative beta (AUC 0.86, 95% CI 0.75–0.98). (**B**) relative alpha (AUC 0.71, 95% CI 0.56–0.86). (**C**) relative theta (AUC 0.71, 95% CI 0.57–0.86). EEG = electroencephalogram, FE = failed extubation, ROC = receiver operating characteristic SE = successful extubation.

Youden´s Index identified the optimal cut off for predicting extubation failure at a beta CV of 0.77 over all channels, which indicates a fluctuation of ± 77% around the mean power value (AUC 0.86, 95% CI 0.75–0.98, sensitivity 92%, specificity 83%, positive predictive value 61%, negative predictive value 97%, Fig. 2).

Logistic regression of relative power values over all channels including duration of MV revealed fluctuation of relative beta as the only independent predictive factor (*p* = 0.004, Table 3). ROC of logistic regression model showed a high discrimination (AUC 0.89).

**Table 3:** Power value fluctuations in patients with successful extubation (SE) versus extubation failure (FE); logistic regression considering coefficients of variation (CV) over all channels.

	Estimate	Std. error	Z value	P value
(Intercept)	7.93	2.50	3.17	**0.002**
Relative beta	− 5.94	2.06	− 2.88	**0.004**
Relative alpha	12.58	8.83	1.42	0.154
Relative theta	4.56	5.34	0.85	0.393
Relative delta	7.38	5.34	0.68	0.494
Alpha delta ratio	− 15.31	7.86	− 1.95	0.051
MV duration	< − 0.01	< 0.01	− 1.91	0.056

MV = mechanical ventilation, Std. error = standard error.

Significant values are in [bold].

## Discussion

Studies on EEG monitoring in NICU patients during the weaning process are sparse. The current prospective study addressed this gap and showed that EEG monitoring in the hours prior to extubation revealed significant differences in patients with successful extubation compared to those with extubation failure.

Successfully extubated patients showed more stable EEG power values over all channels in the relative beta, alpha and theta frequency bands in the hours before extubation with relative beta as an independent predictive factor in multivariate analysis. In contrast, high fluctuations in qEEG before MV withdrawal, particularly a coefficient of variation greater than 0.77 in the beta frequency band, were followed by a higher rate of complications after extubation in this cohort, leading to NIV, reintubation or death. These effects were most pronounced in the fronto-central EEG channels.

NICU patients who are estimated to be ready for extubation as judged by standard cardiovascular parameters, often struggle with insufficient vigilance and breathing drive after extubation, ultimately leading to reintubation. Our data suggest that patients who fail extubation experience greater fluctuations in their levels of consciousness before extubation which is paralleled by greater qEEG fluctuations in the beta and alpha frequency band. Beta/alpha activity better reflects alertness and intact brain function than theta/delta activity^32^. Therefore, it is hypothesized that fluctuations in these frequency bands represent a good marker for vigilance-associated complications after extubation.

During more vigilant phases with better EEG before extubation, FE patients may pass standard assessment for extubation so that extubation is scheduled. However, continuing fluctuations, which may have been clinically less evident before extubation lead to intermittent deterioration of the patients’ condition after extubation resulting in reintubation. This behavior may also explain the result of our study that qEEG fluctuations were more sensitive in differentiating SE form FE patients than an averaged measurement of qEEG frequencies over time.

The EEG power alterations seen in this cohort were most pronounced in the fronto-central channels. This is in line with the findings of previous functional neuroimaging and EEG studies. These studies showed the frontal area to be a surrogate marker for preserved consciousness and responsiveness to extern stimuli in patients without disorders of consciousness^33,34^.

In line with our results, there already are several studies applying EEG and qEEG to patients under MV^35^. In a previous study with 44 MV patients of general intensive care units, higher levels of wakefulness indicated by polysomnography using two central EEG electrodes were observed in patients who successfully passed a SBT assessment and consequently were successfully extubated^36^. However, this study did not include patients with brain disease. Moreover, for monitoring of alertness and sedation level, bispectral index (BIS) monitors are increasingly applied during anesthesia. Yet, in mechanically ventilated ICU patients, discordance between BIS values and clinical sedation was frequently observed, which may point to the need of more than two EEG channels to validly analyze EEG activity in these patients^37,38^. Another approach was followed by Raux et al.^39^, who assessed occurrence of EEG premotor potentials in healthy individuals during induced “ventilator fighting” as a marker of patient-ventilator asynchrony and respiratory discomfort. Thus, 80 EEG epochs of 2.5 s derived from a central needle electrode (Cz) were averaged and assessed. Such an approach requires automated and averaging EEG techniques and is not easily to be applied in routine assessment, but could provide an additional central index of patient–ventilator asynchronies. It is left to future studies how robust and valid this technique is when applied to NICU patients.

As neurocritical patients are a typical population with neurological dysfunction of motor and sensor from the central, future studies could assess whether there are specific abnormal regions in brain and their EEG patterns associated with extubation success and failure. Especially associations to motor responses and/or vigilance should be assessed. This information might contribute to understanding the role of central breathing drive in neurocritical patients.

This study suffers from several limitations.

Above all, only a small cohort of patients could be included and complications after extubation were only observed in 12 patients, including reintubation in 6 cases. Consequently, validity of multivariate logistic regression analysis is weak and no further subgroup analysis, e.g. respecting different pathophysiological aspects of extubation failure, could be performed.

Patient groups differed regarding the duration of MV before extubation. Patients with complications after extubation had been ventilated for a longer period of time, which is in line with the results of prior extubation failure studies^40,41^. Nevertheless, duration of MV was no independent predictive factor in our multivariate analysis.

Moreover, this study cohort included a large portion of patients after seizures and status epilepticus because EEG monitoring is routinely performed in these patients. This could have biased the total patient cohort.

In addition, we cannot exclude effects of medication on EEG analysis. Especially the results regarding the beta frequency band might have been influenced by administered anesthetics, such as midazolam. However, both groups of patients were equally treated by our standard SOP for MV so that choice and dosage of sedative agents were similar in SE and FE patients.

Another limitation of our study is the lack of EEG data after extubation because monitoring was terminated upon extubation based on the decision of the treating physician. EEG monitoring between extubation and re-establishment of ventilation support would have provided the opportunity to analyze cerebral activity in the critical time of spontaneous breathing.

Moreover, additional exploration of qEEG trends during the hours of monitoring may have resulted in identification of a certain EEG pattern which allows for establishing a time point of successful extubation.

Our approach requires EEG monitoring capacity and at least 6 h of stable qEEG analysis, which represents a need for human and technical resources. Yet, EEG monitoring is becoming more and more available, especially on NICUs, and can be maintained for several hours or even days. Reduced montages and quantitative assessment of data are increasingly used for various investigations, as shown by previous studies^24,25^. Current technical advantages even allow for “online” analysis of data, so that clinicians benefit from the results at bedside.

Nevertheless, EEG based risk stratification is obviously not sufficient alone in identifying all patients who are at risk of extubation failure, as dysphagia, low pharyngeal muscle tone and weak cough do also play an important role^42^. Yet, especially neurocritical patients struggle with impaired and variable consciousness, which may be monitored by EEG studies. It is left to future prospective studies to confirm and extent the explorative results of this study in a larger cohort of NICU patients.

## Conclusion

Weaning and extubation management in neurocritical patients is a major challenge for neurointensivists. As standard extubation criteria are frequently not helpful in this patient group, there is a need for further research and innovative measures to address the specific characteristics of neurocritical patients. The ultimate goal is to generate a valid assessment which prevents both delayed and premature extubation. In this regard, EEG can provide valuable additional information, especially when it comes to the question of sufficient vigilance and respiratory drive in the weaning process. The results of this study suggest that especially the stability of quantitative EEG power values in the hours before extubation could represent a non-invasive, examiner-independent indicator to help treating (NICU) physicians finding the optimal time-point to disconnect the patient from the ventilator.

## Data Availability

The datasets used and/or analysed during the current study are available from the corresponding author on reasonable request.

## Abbreviations

ADR: Alpha delta ratio
ASB: Assisted spontaneous breathing
AUC: Area under the curve
BIS: Bispectral index
CPAP: Continuous positive airway pressure
CV: Coefficient of variation
FE: Failed extubation
ICP: Intracranial pressure
MV: Mechanical ventilation
NICU: Neurological intensive care unit
NIV: Non-invasive ventilation
PEEP: Positive end-expiratory pressure
Psupp: ASB Support
RA: Relative alpha
RASS: Richmond Agitation Sedation Scale
RB: Relative beta
RD: Relative delta
RSBI: Rapid shallow breathing index
RT: Relative theta
SE: Successful extubation
SBT: Spontaneous breathing trial
SOP: Standard operating procedure
qEEG: Quantitative electroencephalography
